# Functional Characterization of an Anthocyanin Dimalonyltransferase in Maize

**DOI:** 10.3390/molecules26072020

**Published:** 2021-04-01

**Authors:** Michael Paulsmeyer, John Juvik

**Affiliations:** Department of Crop Sciences, University of Illinois at Urbana-Champaign, Urbana, IL 61801, USA; juvik@illinois.edu

**Keywords:** acyltransferase, acylation, cyanidin, enzyme kinetics, malonyltransferase

## Abstract

Anthocyanins are pigments with appealing hues that are currently being used as sources of natural colorants. The interaction of acylation on the stability of anthocyanin molecules has long been known. Maize is an abundant source of malonylglucoside and dimalonylglucoside anthocyanins. The enzyme Aat1 is an anthocyanin acyltransferase known to synthesize the majority of acylated anthocyanins in maize. In this paper, we characterize the substrate specificity and reaction kinetics of Aat1. It was found that Aat1 has anthocyanin 3-*O*-glucoside dimalonyltransferase activity and is only the second enzyme of this type characterized to this date. Our results indicate that Aat1 can utilize malonyl-CoA; succinyl-CoA and every anthocyanin 3-*O*-glucoside tested. Results of this study provide insight into the structure–function relations of dimalonyltransferases and give a unique insight into the activity of monocot anthocyanin acyltransferases.

## 1. Introduction

Anthocyanins are a diverse group of pigments that are ubiquitous in the plant kingdom. They impart most of the orange to red and purple to blue colors present in plant species. Due to the prevalence of these compounds in many plant species and due to the attractiveness of their hues, anthocyanins are becoming popular natural substitutes for synthetic dyes. Purple corn is becoming an increasingly popular choice for anthocyanin extraction. The economy of scale for purple corn and the utilization of anthocyanins as co-products in the maize supply chain make purple corn an attractive alternative to other natural colorant sources currently on the market [1,2]. Anthocyanins are also well-known health-promoting compounds. Purple corn extracts have a much higher antioxidant capacity than any other fruit and vegetable extract [3]. In a review of the literature, purple corn extracts are shown to have associations with anti-inflammatory, antimutagenic, anticarcinogenic, and anti-angiogenesis [4]. Incorporation of maize extracts as sources of natural colorants will have dietary implications for increasing human health.

A major obstacle for adopting anthocyanins as natural colorants, however, is the inferior stability compared to synthetic dyes. Stability of anthocyanins is controlled in part by the inherent structure of the molecule. Adding acyl groups to the anthocyanin glycone is one method plants use to enhance the stability of these pigments. Acylation is defined as the esterification of organic acids to the sugar of the anthocyanin molecule. Two types of acylation are possible: aliphatic and aromatic. Aromatic acylation generally confers the greatest stability probably due to intramolecular stacking of pigment molecules [5]. Aliphatic acylation has also been shown to be important as it enhances total anthocyanin content in maize [6]. Aliphatic acylation has also been shown to decrease enzymatic degradation and increase the uptake of anthocyanins into the vacuole, all while maintaining the same hues of the molecule [7,8]. In vitro studies show that acylation protects the anthocyanin molecule during heat stress, intense light, and high pH [5,9]. Manipulating proportions of acylated anthocyanins in extracts seems to be an effective way of enhancing stability of natural colorants.

**Figure 1:**
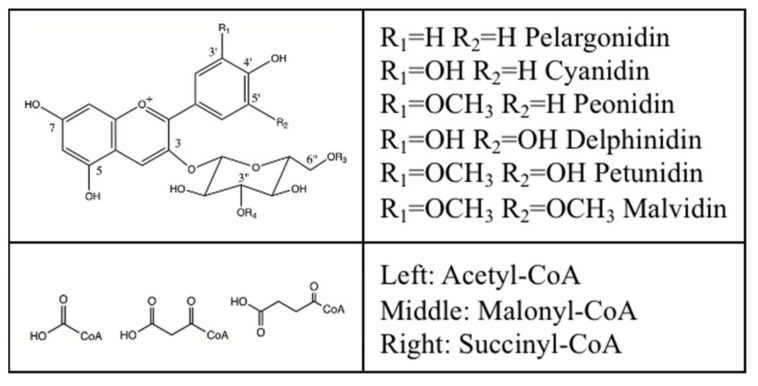
Structure of an anthocyanin 3-*O*-glucoside molecule with common substitutions.

In maize, acylated anthocyanins account for a majority of the total anthocyanins produced, with aliphatic malonic acid derivatives being the most represented form [1]. In particular, the 6”-position of the glucoside is the first acylated position followed by the 3”-position for dimalonylglucosides (Figure 1). An enzyme catalyzing this reaction in maize has been discovered and is named *Anthocyanin acyltransferase1* (*Aat1*) [6]. Nearly all the acylated anthocyanins were depleted in dysfunctional mutants of this gene. However, it is unknown if the enzyme itself is capable of producing dimalonylglucoside anthocyanins present in the maize grain or what sort of specificity the enzyme has in terms of substrates. Only one enzyme has been characterized to this date that has dimalonyltransferase activity—chrysanthemum (*Dendranthema* × *morifolium*) Dm3Mat2 [10]. In this study, we characterize the activity of recombinant Aat1 and determine specificity and reaction kinetics.

## 2. Results

### 2.1. Phylogenetic Analysis

**Figure 2:**
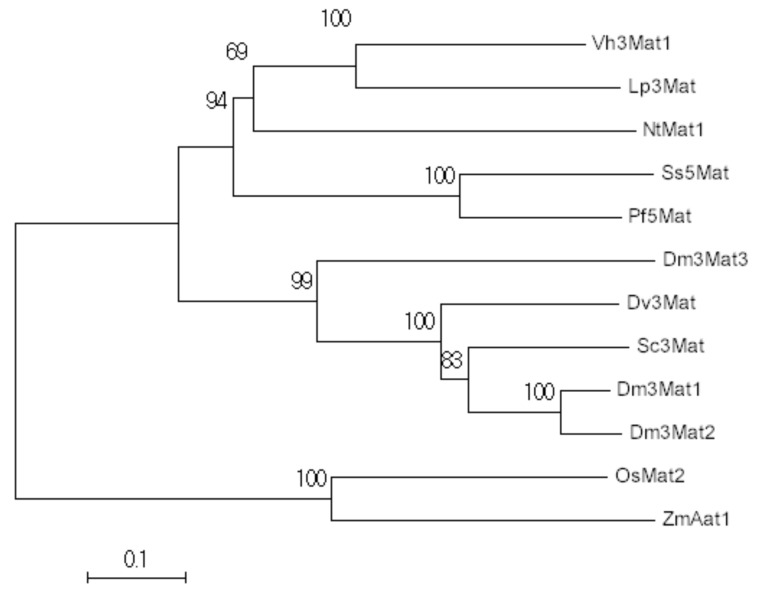
Phylogenetic analysis of flavonoid malonyltransferases. From top to bottom: *Glandularia* × *hybrida* Vh3Mat1 (AAS77402.1), *Lamium purpureum* Lp3Mat (AAS77404.1), *Nicotiana tabacum* NtMat1 (BAD93691.1), *Salvia splendens* Ss5Mat (AAL50566.1), *Perilla frutescens* Pf5Mat (AAL50565.1), *Chrysanthemum* × *morifolium* Dm3Mat3 (BAF50706.1), *Dahlia pinnata* Dv3Mat (Q8GSN8.1), *Pericallis cruenta* Sc3Mat (AAO38058.1), *Chrysanthemum* × *morifolium* Dm3Mat1 (AAQ63615.1), *Chrysanthemum* × *morifolium* Dm3Mat2 (AAQ63616.1), *Oryza sativa* OsMat1 (NP_001046855.1), *Zea mays* ZmAat1 (NP_001148286.2). The evolutionary history was inferred using the Neighbor-Joining method [17]. The percentage of replicate trees in which the associated taxa clustered together in the bootstrap test (10,000 replicates) are shown next to the branches [18]. The tree is drawn to scale, with branch lengths in the same units as those of the evolutionary distances used to infer the phylogenetic tree. The evolutionary distances were computed using the Poisson correction method [19]. Evolutionary analyses were conducted in MEGA7 [20].

Phylogenetic analysis of known flavonoid malonyltransferases is similar to previously reported analyses with other acyltransferase enzymes (Figure 2). Another monocot flavonoid malonyltransferase in *Oryza sativa*, OsMat2, is most similar to Aat1 in maize and shares a clade in the phylogenetic tree. However, they only share 51% sequence identity. Maize Aat1 shares 28% to 31% identity with other malonyltransferases in eudicots, which is typical of acyltransferase members. Previous studies report that the minimal amount of sequence similarity among acyltransferases is 25% to 34% [11]. Despite this high sequence diversity, flavonoid acyltransferases share unique signatures that are responsible for catalytic activity. The His-X-X-X-Asp domain (Motif 1) is a motif found in every acyltransferase member (Supplementary Materials, Figure S1). Through protein crystallography, it was found that the His-residue in particular is responsible for catalyzing the acyl transfer [12,13]. The kinetic mechanism for anthocyanin malonyltransferases is most likely via a ternary complex in which the His residue from Motif 1 deprotonates the hydroxyl group of the acyl acceptor initiating a nucleophilic attack on the thioester of the acyl donor [14]. Motifs Tyr-[Phe/Lys]-Gly Asn-Cys (Motif 2) and Asp-Phe-Gly [Trp/Phe]-Gly (Motif 3) are anthocyanin specific (Figure S1). Not many flavonoid acyltransferases have been characterized in monocots. The only other represented member is OsMat2, which has no activity with anthocyanin substrates [15]. Both Aat1 and OsMat2 share the Phe variant of Motif 3, while all eudicot flavonoid acyltransferases contain the Trp residue (Figure S1). More monocot acyltransferases need to be characterized to test whether this variant is shared among the monocot lineage. As for the Motif 2 variant present in maize, it was proposed in Suzuki et al. (2004) that the Lys substitution may be involved with dimalonyltransferase activity as it may alter the binding pocket [10]. Manjasetty et al. (2012) confirm in a crystallographic study that Motif 2 does in fact shape the binding site of the acyl donor [12]. However, OsMat2 also contains the Lys substitution, but does not have dimalonyltransferase activity. Unlike other flavonoid malonyltransferases, however, OsMat2 is able to freely accept both 3-*O*-glucoside and 7-*O*-glucoside forms equally, which is unique for a flavonoid malonyltransferase [16]. Future studies should focus on the effect the Lys substitution has on the binding pocket and dimalonyltransferase activity.

### 2.2. Characterization of Aat1 Recombinant Protein

**Figure 3:**
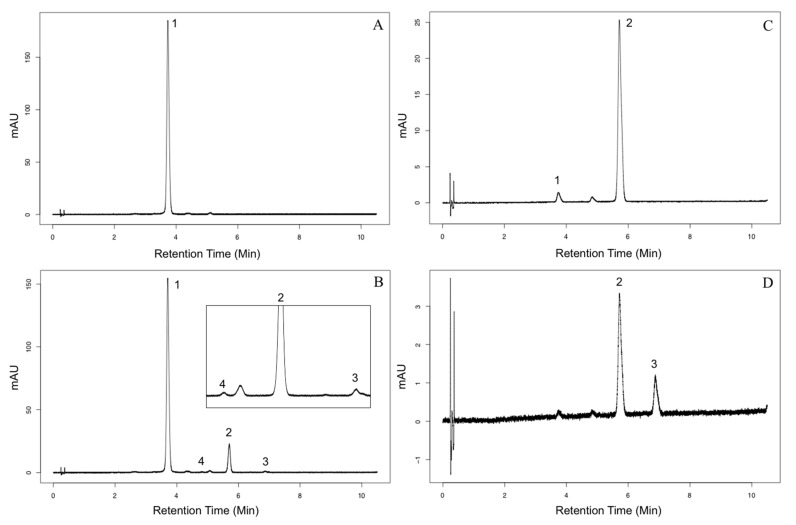
Aat1 is capable of synthesizing dimalonylglucoside anthocyanins. (**A**) Cyanidin 3-*O*-glucoside standard (Extrasynthese), (**B**) enzyme reaction with cyanidin 3-*O*-glucoside with enzyme products expanded inset. (**C**) Cyanidin 3-*O*-6”-malonylglucoside semi-preparative HPLC fraction. (**D**) Enzyme reaction with cyanidin 3-*O*-6”-malonylglucoside. Compound identities: (1) Cyanidin 3-*O*-glucoside, (2) Cyanidin 3-*O*-6”-malonylglucoside, (3) Cyanidin 3-*O*-3”,6”-dimalonylglucoside, (4) Cyanidin 3-*O*-3”-malonylglucoside.

The gene model for *Aat1* has been revised since it was first discovered [6]. The revised transcript adds 94 amino acids missing due to an incorrectly annotated transcription start site. The corrected transcript was inserted into a pET-30a(+) 6x-His-tag expression vector and transformed into Rosetta-gami™ 2 cells (Millipore Sigma, Burlington, MA, USA), which alleviates codon bias and enhances disulfide bond formation for eukaryotic proteins. Protein was purified to apparent purity after affinity tag purification (Figure S2). The protein showed strong malonyltransferase activity and could catalyze the malonyl transfer to the 6”-position of the 3-*O*-glucoside. In addition, maize Aat1 can catalyze the subsequent addition of a malonyl group to form dimalonylglucosides (Figure 3). This makes Aat1 only the second anthocyanin dimalonyltransferase characterized to this date. The formation of 3”-malonylglucosides is possible, but only detected in very small amounts (Figure 3). The dimalonyltransferase reaction for Aat1 occurs much more slowly than the initial malonylation reaction (Figure S3), but the specificity constant is slightly higher for cyanidin 3-*O*-6”-malonylglucoside than the glucoside form (Table 1). The enzyme has an activity range between a pH of 5.5 and 10 with a pH between 6.6 and 8.0 being maximal. Although not explicitly tested here, other anthocyanin malonyltransferases are inhibited by the presence of Cd^2+^, Cu^2+^, Fe^2+^, Hg^2+^, Mg^2+^, and Zn^2+^ [7,21,22]. Future studies should investigate the effect of these cations on Aat1 enzyme function.

Specificity and reaction kinetics of Aat1 varied widely from the other anthocyanin dimalonyltransferase, Dm3Mat2 [10]. The most striking difference is the apparently slow rate of enzyme activity (Figure S3). The estimate for K_cat_ ranged from 7.25 × 10^−4^ to 6.23 × 10^−3^·s^−1^ (Table 1). This is in comparison with Dm3Mat2 which had rates ranging from 0.27 to 11.1 s^−1^ [10]. The slow turnover rate may be the reason a typical blue corn sample does not completely convert all available 3-*O-*glucoside forms to malonylglucosides. In a survey of 98 diverse blue corn varieties, the average percentage of acylation was 63.19% [1]. This is in comparison to chrysanthemum, which has nearly 100% acylated anthocyanins present in the flowers [23]. In addition, Aat1 differs in that it cannot effectively utilize delphinidin 3-*O*-glucoside as well as other 3-*O*-glucoside anthocyanins. No maize lines to this date have been found to produce appreciable amounts of delphinidin, malvidin, or petunidin, especially in the grain [1]. Cyanidin 3-*O*-glucoside appears to be the most preferred substrate for Aat1, but not in Dm3Mat2 [10]. Similar to other anthocyanin acyltransferases, Aat1 can utilize quercetin 3-*O*-glucoside (results not shown) [7,10]. Moreover, the enzyme can utilize succinyl-CoA as an acyl acceptor, but not acetyl-CoA. Acetyl-CoA may actually be an inhibitor of enzyme function according to other studies [7,21,22]. The turnover rate and specificity constant for succinyl-CoA was much lower than that for malonyl-CoA, enforcing the notion that the enzyme is indeed a malonyltransferase (Table 1). Moreover, the enzyme cannot utilize rutinoside or 3,5-diglucoside anthocyanins (data not shown) probably due to steric hinderance of the additional sugars in the binding pocket. 

## 3. Materials and Methods

### 3.1. Substrates

Analytical standards of cyanidin 3-*O*-glucoside, delphinidin 3-*O*-glucoside, pelargonidin 3-*O*-glucoside, and peonidin 3-*O*-glucoside were purchased from Extrasynthese (Genay, France). Malonyl-CoA, succinyl-CoA, and acetyl-CoA were purchased from CoALA Biosciences (Austin, TX, USA). Quercetin 3-*O*-glucoside standard was purchased from Sigma-Aldrich. Anthocyanins were reconstituted in 0.1% HCl in water, while acyl donor substrates were reconstituted in water and used within a day. To prepare malonylglucoside standards, an enzyme assay (see Section 3.4 *Enzyme Assays*) was performed under standard conditions and run on a semi-preparative HPLC (see Section 3.5 *Liquid Chromatography and Mass Spectrometry*) to isolate the malonylglucoside peak. The purified aliquots were combined, concentrated in a SpeedVac^®^ AES2010 concentrator (Thermo Savant, Waltham, MA, USA) at 43 °C and then lyophilized. Powder was reconstituted with 0.1% HCl in water and the concentration was adjusted to 600 µM based on the A520 of cyanidin 3-*O*-glucoside given that the molar extinction coefficients are similar [7]. Purity at A520 was 91.95% according to the UHPLC protocol described below (see Section 3.5 *Liquid Chromatography and Mass Spectrometry*).

### 3.2. Cloning

Full length *Aat1* transcript (NCBI Reference Sequence NP_001148286.2) was amplified using PCR with primers 5′-CATGATTCGAATTCATGGCGGCAGCAACGGCAACT-3′ and 5′-GCTACGATAAGCTTTCACAGCAACCGGAGCCACTCCA-3′ that introduce *EcoR*I and *Hind*III sites (underlined). Restriction enzyme cloning introduced the transcripts into the pET-30a(+) vector (Millipore Sigma, Burlington, MA, USA) as described by the supplier [24]. Vectors were transformed into chemically competent Rosetta-gami™ 2(DE3)pLysS Competent Cells (Millipore Sigma, Burlington, MA, USA). LB media supplemented with 1 mM MgSO_4_ to assist growth [25] and 50 µg/mL kanamycin for selection were inoculated with cultures stored at −80 °C in 25% (*v/v*) glycerol. After cultivating overnight at 37 °C, two mL of overnight culture was added to 100 mL fresh media and grown at 37 °C to an OD600 of 0.6 to 0.8. Cultures were put on ice for five minutes before being induced with 0.4 mM IPTG overnight at 18 °C. Overnight cultures were centrifuged at 1600× *g* for 10 min. Media were decanted and pellets were resuspended in 100 mM potassium phosphate (pH 8.0), 10% glycerol, 1 mM EDTA, and 5 mM 2-mercaptoethanol (Buffer A) with an addition of 1 mM PMSF. Bacterial lysis by sonication was done on ice with a 20 kHz probe set to 50% power with five 10 s bursts with 20 s rests between pulses. 

### 3.3. Affinity Tag Purification 

To capture 6x-His-tagged recombinant Aat1, one mL HisPur™ Ni-NTA agarose resin (Thermo Fisher Scientific, LLC, Waltham, MA, USA) was equilibrated with 10 column volumes ultrapure water and 20 column volumes of Buffer A + 10 mM imidazole before the addition of the protein extract. Whole crude protein extract was filtered in a 0.45 µm PES syringe filter (Millipore Sigma, Burlington, MA, USA) and equilibrated with constant shaking for 1 hr. The resin with captured protein was washed with 30 column volumes of Buffer A + 10 mM imidazole. The 6x-His-tagged protein was eluted in 3 column volumes of Buffer A + 500 mM imidazole. Purified protein was desalted in a G-25 MidiTrap (Cyteva, Marlborough, MA, USA) desalting column according to manufacturer’s instruction using 100 mM potassium phosphate (pH 8.0) + 10% glycerol as the equilibration buffer. SDS-PAGE analysis was done on a 10% Mini-Protean^®^ TGX™ gel with 50 µL wells (Bio-Rad Laboratories, Inc., Hercules, CA, USA).

### 3.4. Enzyme Assays

A standard reaction consisted of 60 µM malonyl-CoA as the acyl donor and 120 µM cyanidin 3-*O*-glucoside as the acyl acceptor in 100 mM potassium phosphate (pH 8.0). To calculate reaction kinetics, acyl acceptors were varied between 5 and 120 µM, acyl donors were varied between 5 and 30 µM, and cyanidin 3-*O-*6”-malonylglucoside was varied between 2.5 and 30 µM. Anthocyanins, which were diluted with 0.1% HCl in water, were no more than 5% of the reaction volume so as to not change the pH of the reaction. The final reaction volume was 100 µL and each assay was replicated three times. Reactions were run for 30 min at 30 °C before being halted with 100 µL ice cold 4% (*v/v*) formic acid. Enzyme assays determining pH preference used 100 mM sodium acetate (pH 3.0 to 5.5), 100 mM potassium phosphate (pH 6.0 to 8.0), 100 mM Tris (pH 8.5 and 9.0), and 100 mM CAPS (pH 10 and 11). Kinetics constants were determined using the rearranged Michaelis–Menten equation proposed by K. A. Johnson (2019) [26]. Constants K_cat_ and K_sp_ (K_cat_/K_m_) were solved using the “nls” non-linear modeling function in R [27]. Velocity was calculated as the summation of micromoles of product formed over seconds.

### 3.5. Liquid Chromatography and Mass Spectrometry

Anthocyanins in enzyme assays were quantified using an Agilent 1290 series UHPLC. An aliquot of 20 µL of enzyme assay was separated in an Agilent InfinityLab Poroshell 120 SB-C_18_ (4.6 mm × 100 mm, 1.9 µm) column (Santa Clara, CA, USA) kept at 50 °C. The wavelengths used for detection were 520 nm for all anthocyanins or 280 nm for quercetin 3-*O*-glucoside. The mobile phase consisted of 2% formic acid (A) and acetonitrile (B) at a rate of 1.7 mL/min in a gradient from 4% B from 0 min to 1 min, 4% to 20% B from 1 to 10 min, then 20% to 100% in 30 s and back to 4% B in 30 s. The column was equilibrated with 4% B for 4 min between samples. Anthocyanin standards were varied in a linear range to calibrate peak areas. To confirm compounds formed in enzyme assays, representative samples were analyzed on a Waters Synapt G2-Si ESI/LC-MS/MS at the Mass Spectrometry Lab at the University of Illinois (Urbana, IL, USA) using the same column and temperature as the enzyme assays. The gradient was adjusted to run on the machine. The mobile phase consisted of 0.1% formic acid (A) and acetonitrile (B) at a rate of 1 mL/min in a gradient of 4% B from 0 to 1 min, 4% B to 25% B from 1 to 25 min, then a 95% B hold for 5 min and back to equilibration with 4% B for 5 min. Aliquots of 10 µL were injected for each sample. To prepare semi-pure malonylglucoside standards, standard enzyme assays were run on an Agilent 1100 Series HPLC with a semi-preparative fraction collector. Aliquots totaling 75 µL were separated in a Grace Prevail RP-C_18_ (4.6 mm × 250 mm, 5 µm) column (Columbia, MD, USA) heated to 30 °C. The diode array detector was monitored at 280 nm. Mobile phases used were 2% formic acid (A) and acetonitrile (B) with a flowrate of 1 mL/min in a gradient of 10% B at 0 min, 30% B at 35 min, 100% B from 36 to 38 min, and equilibration for 5 min at 10% B.

## 4. Conclusions

Maize Aat1 is an anthocyanin dimalonyltransferase responsible for synthesizing the majority of anthocyanins in maize grain. It can utilize malonyl-CoA, succinyl-CoA and a variety of 3-*O*-glucoside anthocyanin substrates. Future studies should investigate the effect of the residues that may be important for dimalonyltransferase activity, especially the Lys substitution in Motif 2 (Figure S1). In addition, more malonyltransferase members in other monocot species need to be explored to determine phylogenetic relationships and structure–activity relationships. Overall, investigations into Aat1 have implications on increasing anthocyanin content in the grain and therefore implications on human health. Stabilizing anthocyanins, especially in purple and blue corn, has implications on making more economical sources of natural colorants and increasing the health-promoting aspects of maize. 

## Figures and Tables

**Table 1:** Kinetic parameters and substrate specificity of Aat1.

Compound	Relative Activity ^a^	K_m_	K_cat_	K_cat_/K_m_
Units	%	µM	s^−1^ × 1000	µM^−1^·s^−1^ × 1000
Acyl Acceptors ^b^				
Cyanidin 3-*O*-Glucoside	100 ± 2.07	13.06 ± 0.23	6.233 ± 0.145	0.477 ± 0.034
Pelargonidin 3-*O*-Glucoside	89.8 ± 3.24	8.92 ± 0.41	5.594 ± 0.074	0.627 ± 0.030
Peonidin 3-*O*-Glucoside	43 ± 1.55	5.14 ± 0.93	2.596 ± 0.028	0.505 ± 0.026
Delphinidin 3-*O*-Glucoside	20.1 ± 0.74	ND	ND	ND
Cyanidin 3-*O*-6”-Malonylglucoside	ND	1.33 ± 3.97	0.725 ± 0.044	0.543 ± 0.173
Acyl Donors ^c^				
Malonyl-CoA	100 ± 2.07	40.12 ± 0.04	8.043 ± 0.751	0.200 ± 0.026
Succinyl-CoA	18.5 ± 0.67	60.09 ± 0.02	1.607 ± 0.194	0.027 ± 0.004
Acetyl-CoA	<0.1	ND	ND	ND

^a^ Relative activities were calculated with acyl donors in a final concentration of 60 µM and acyl acceptors in a final concentration of 120 µM. The reaction with malonyl-CoA and cyanidin 3-*O*-glucoside was taken to be 100%. ^b^ Malonyl-CoA (60 µM) was the acyl donor. ^c^ Cyanidin 3-*O*-glucoside (120 µM) was the acyl acceptor. ND = Not Determined; reported values are ± standard error.

## Data Availability

Data is available upon request by the corresponding author.

## Supplementary Materials

The following are available online: Figure S1. Alignment of known anthocyanin malonyltransferases; Figure S2. Purification of Recombinant Aat1; Figure S3. Michaelis–Menten Plots.

## Sample Availability

Plasmids and bacteria strains are available upon request from the authors.
