# Supplementary material for: Functional Characterization of an Anthocyanin Dimalonyltransferase in Maize
Source: Molecules. 2021 Apr 1;26(7):2020. doi: 10.3390/molecules26072020 (PMC8037723; doi:10.3390/molecules26072020)
Supplement: Supplementary file 1 [file molecules-26-02020-s001.pdf]

Supplementary Material

Figure S1: Alignment of known anthocyanin malonyltransferases. From top to bottom: *Glandularia × hybrida* Vh3Mat1 (AAS77402.1), *Lamium purpureum* Lp3Mat (AAS77404.1), *Nicotiana tabacum* NtMat1 (BAD93691.1), *Salvia splendens* Ss5Mat (AAL50566.1), *Dahlia pinnata* Dv3Mat (Q8GSN8.1), *Pericallis cruenta* Sc3Mat (AAO38058.1), *Perilla frutescens* Pf5Mat (AAL50565.1), *Chrysanthemum × morifolium* Dm3Mat1 (AAQ63615.1), *Chrysanthemum × morifolium* Dm3Mat2 (AAQ63616.1), *Chrysanthemum × morifolium* Dm3Mat3 (BAF50706.1), *Oryza sativa* OsMat1 (NP_001046855.1), *Zea mays* ZmAat1 (NP_001148286.2). Motifs 1 to 3 are indicated above their respective amino acids.

Figure S2: Purification of Recombinant Aat1. 1) Uninduced culture, 2) resuspended culture before sonication, 3) soluble protein fraction after sonication, 4) flow-through of the Ni-NTA column, 5) purified Aat1 with an arrow indicating the protein band.

Figure S3: Michaelis-Menten Plots. Dots indicate measured velocity over initial substrate concentration. Velocity was determined as micromoles of product formed over time. Red lines represent the fitted model as determined by the Michael-Menten equation modified by K. A. Johnson (2019) [1].

References

Figure S1: Alignment of known anthocyanin malonyltransferases. From top to bottom: *Glandularia × hybrida* Vh3Mat1 (AAS77402.1), *Lamium purpureum* Lp3Mat (AAS77404.1), *Nicotiana tabacum* NtMat1 (BAD93691.1), *Salvia splendens* Ss5Mat (AAL50566.1), *Dahlia pinnata* Dv3Mat (Q8GSN8.1), *Pericallis cruenta* Sc3Mat (AAO38058.1), *Perilla frutescens* Pf5Mat (AAL50565.1), *Chrysanthemum × morifolium* Dm3Mat1 (AAQ63615.1), *Chrysanthemum × morifolium* Dm3Mat2 (AAQ63616.1), *Chrysanthemum × morifolium* Dm3Mat3 (BAF50706.1), *Oryza sativa* OsMat1 (NP_001046855.1), *Zea mays* ZmAat1 (NP_001148286.2). Motifs 1 to 3 are indicated above their respective amino acids.

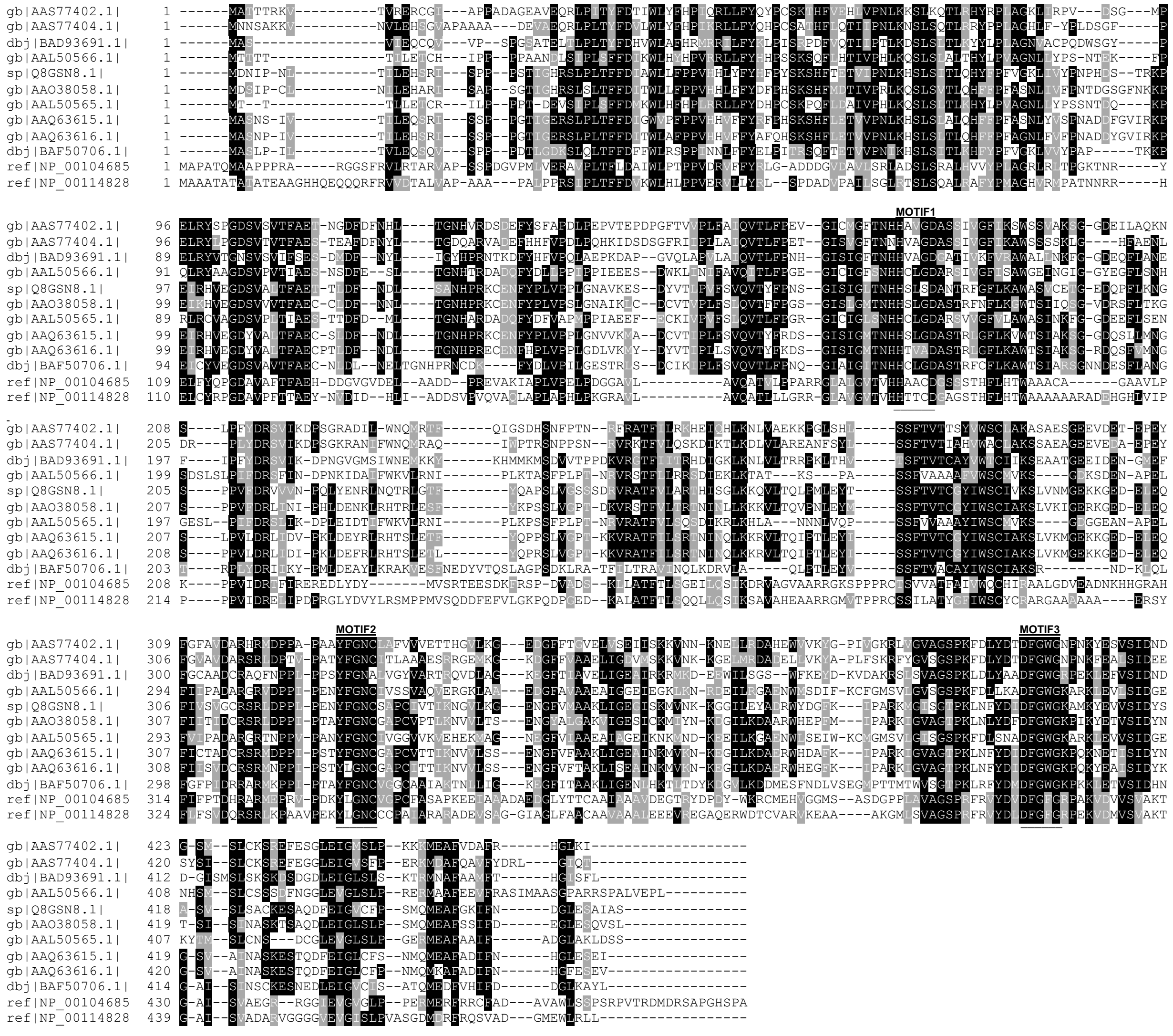

Figure S2: Purification of Recombinant Aat1. 1) Uninduced culture, 2) resuspended culture before sonication, 3) soluble protein fraction after sonication, 4) flow-through of the Ni-NTA column, 5) purified Aat1 with an arrow indicating the protein band.

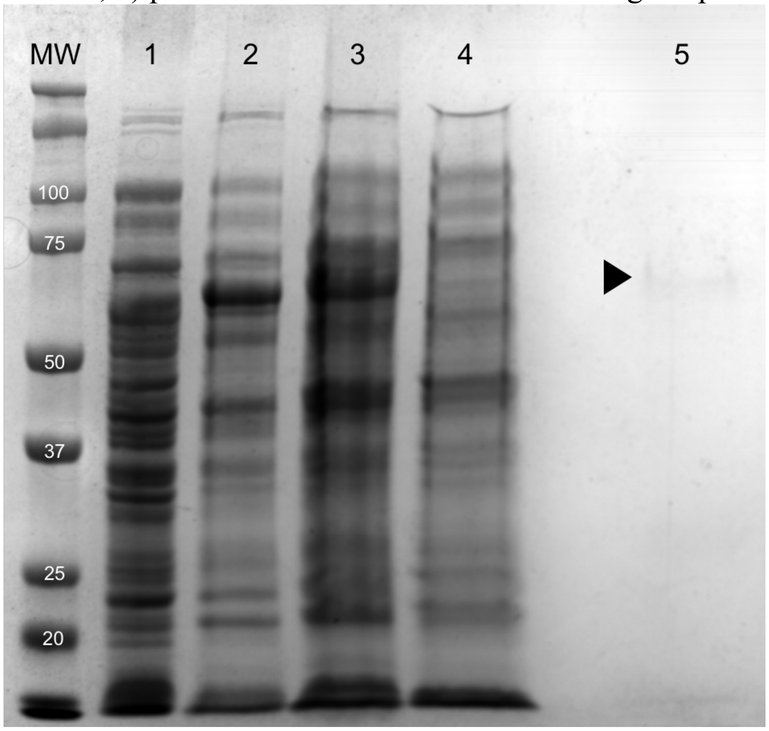

Figure S3: Michaelis-Menten Plots. Dots indicate measured velocity over initial substrate concentration. Velocity was determined as micromoles of product formed over time. Red lines represent the fitted model as determined by the Michael-Menten equation modified by K.A. Johnson [1].

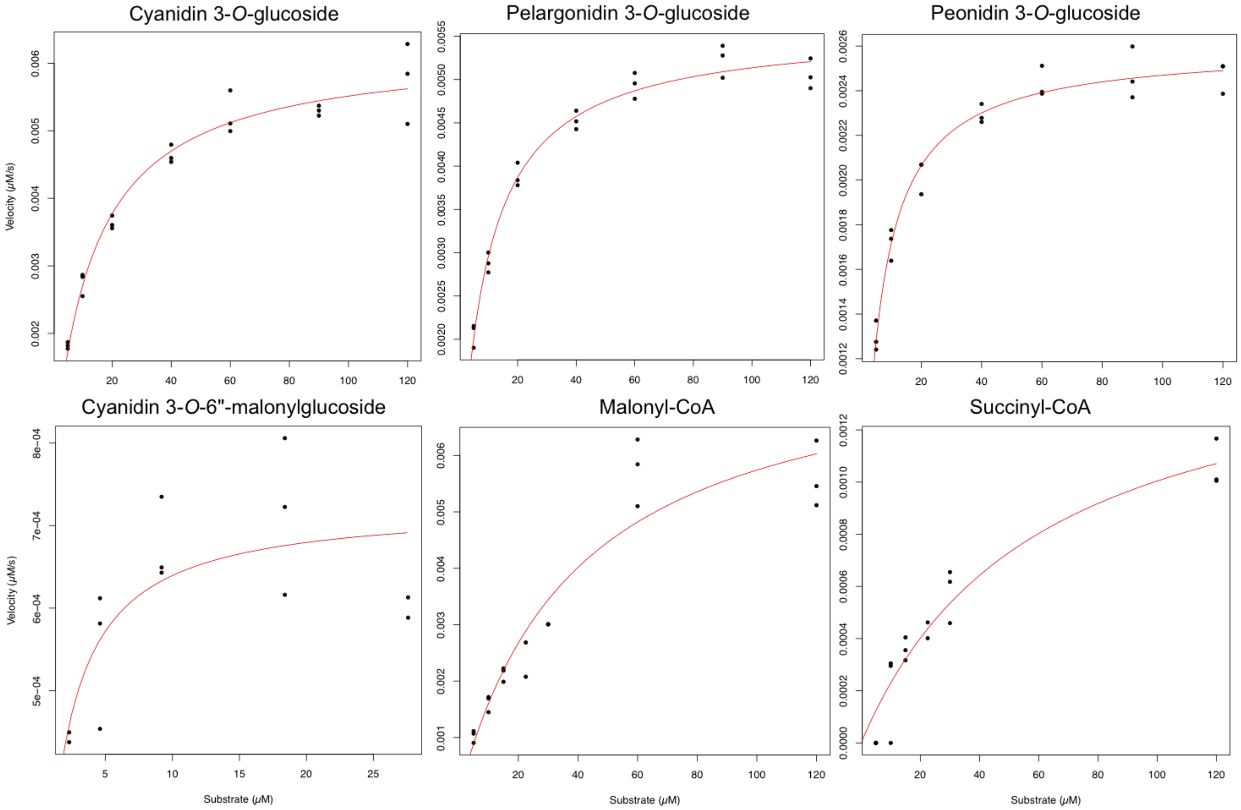

References:

1. Johnson, K.A. New Standards for Collecting and Fitting Steady State Kinetic Data. Beilstein J. Org. Chem. 2019, 15, 16–29, doi:10.3762/bjoc.15.2.
